# Epigenetic Regulations in Neural Stem Cells and Neurological Diseases

**DOI:** 10.1155/2018/6087143

**Published:** 2018-03-18

**Authors:** Hang Zhou, Bin Wang, Hao Sun, Xingshun Xu, Yongxiang Wang

**Affiliations:** ^1^Department of Neurology, The Second Affiliated Hospital of Soochow University, Suzhou, China; ^2^Institute of Neuroscience, Soochow University, Suzhou, China; ^3^Department of Orthopedics, Clinical Medical School, Yangzhou University, Northern Jiangsu People's Hospital, Yangzhou 225001, China

## Abstract

Among the regulatory mechanisms of the renewal and differentiation of neural stem cells, recent evidences support that epigenetic modifications such as DNA methylation, histone modification, and noncoding RNAs play critical roles in the regulation on the proliferation and differentiation of neural stem cells. In this review, we discussed recent advances of DNA modifications on the regulative mechanisms of neural stem cells. Among these epigenetic modifications, DNA 5-hydroxymethylcytosine (5hmC) modification is emerging as an important modulator on the proliferation and differentiation of neural stem cells. At the same time, Ten-eleven translocation (Tet) methylcytosine dioxygenases, the rate-limiting enzyme for the 5-hydroxymethylation reaction from 5-methylcytosine to 5-hydroxymethylcytosine, play a critical role in the tumorigenesis and the proliferation and differentiation of stem cells. The functions of 5hmC and TET proteins on neural stem cells and their roles in neurological diseases are discussed.

## 1. Introduction

Human beings are developed from a fertilized egg into a complete individual; during the whole process, a series of precise regulations on the development are included, such as gene expression and gene silence [1], transcriptional regulation [2], posttranscriptional regulation [3], hormone regulation [4], chromosome behavior regulation [5], and apoptosis [6]. For these different regulative pathways, their target cells are embryonic stem cells (ESCs). ESCs are totipotent stem cells that had a capability to proliferate and differentiate into appropriate lineages to form specialized cells and organs and play a central role in the developmental process [7]. Due to the powerful plasticity and potential of ESCs as a high potential cell replacement therapy for many diseases, stem cells are considered to have an appreciable translational prospect in the field of regenerative medicine [8]. Except for ESCs at the embryonic stage of the development, adult stem cells (ASCs) exist in different tissues at the adult stage of the development [9]. ASCs are often in a resting state in individuals and exhibit different potentials of regeneration and differentiation under pathological conditions or special incentives. Reynolds and Weiss first found that the neurons isolated from the striatum of the adult mouse brain could proliferate and differentiate in vitro with epidermal growth factors [9], indicating the existence of neural stem cells (NSCs) in the mature nervous system. They also demonstrated that NSC has the ability to self-renew and differentiate into other types of cells like neurons, astrocytes, and oligodendrocytes under many conditions such as growth factors, neurotransmitters, hormones, injury, or environmental factors [9]. However, the renewal and differentiation ability of NSC is limited; in the process of aging or pathological conditions, neuronal cell loss is much more than newly generated neurons and glial cells from NSCs, resulting in different neurological disorders including Alzheimer's disease [10], Parkinson's disease [11], Huntington's disease [12], neuroendocrine tumors [13], and ataxia [14]. Therefore, the regulation on the renewal and differentiation of NSCs or NSC transplantation therapy are considered an important therapeutic strategy for the treatment of these neurodegenerative diseases.

Among the regulatory mechanisms of the renewal and differentiation of NSCs, epigenetic modification plays a critical role in monitoring the phase transition during individual development, maintaining the directional differentiation of stem cells, regulating the proliferation of specific cells, and controlling the process of differentiation [15, 16]. For example, in the process of umbilical cord mesenchymal stem cells (UMSCs) being differentiated to neural stem-like cells (uNSCLs), E1A-like inhibitor of differentiation 3 (EID3), an important member of EID gene family that has the main function of p300/CBP inhibitors (a transcriptional coactivator) in response to cell transformation, growth arrest, or cell apoptosis, directly interacts with DNMT3A, a DNA methyltransferase (DNMT) for DNA methylation, suggesting that DNA methylation may be involved the regulation of transdifferentiating from UMSCs to uNSCLs as a key mechanism in epigenetic regulation of stem cell reprogramming [17]. So far, epigenetic modification is a hot topic in recent years. Except for DNA methylation, histone modification, micro-RNA, chromatin remodeling, and other epigenetic modification are found to play important roles in the regulation of stem cells [18]. In this article, we will review the recent advances of different epigenetic modifications on NSCs, but mainly focus on the role of 5hmC as a new player in the regulation of the renewal and differentiation of ESCs or NSCs.

## 2. Recent Advances on Epigenetic Regulation on Stem Cells

It is strongly believed that the basis of cell differentiation in ontogeny is based on the regulation of intracellular factors, while environmental factors also play a role as a main cause [19]. Epigenetic modifications including methylation, acetylation, ubiquitination, and phosphorylation on DNA, RNA, or proteins mediate the interaction between the environment and the organism [20]. Interestingly, recent evidences demonstrate that epigenetic modification changes can be inherited to the next generation [21]. Here, we present a brief overview of current advances on epigenetic modifications and NSCs.

### 2.1. DNA Methylation

The increasing evidences demonstrate that DNA methylation is involved in the proliferation and differentiation of stem cells [22]. DNA methylation prevents transcriptional factors from binding to promoters, such as Oct4 and Nanog, thereby limiting gene expression [23]. The process of DNA methylation is catalyzed by DNA methyltransferase, mainly DNMT1, DNMT3A, and DNMT3B. DNMT3 enzyme is a de novo methyltransferase [24] and DNMT1 is mainly involved in the maintaining of DNA methylation in dividing somatic cells [25]. The deletion of DNMT3A in hematopoietic stem cells impaired the differentiation of transplanted hematopoietic stem cells and increased the level of hematopoietic stem cells in the bone marrow [22]. In skeletal muscle stem cells, the DNA methylation of CpG dinucleotide in the promoter or enhancer region reduces gene expression of Pax7 and MyoD [26]. Similarly, Uhrf1 (ubiquitin-like PHD ring finger-1; also known as Np95) mainly interacts with DNMT1 to maintain DNA methylation in NSCs; the deletion of Uhrf1 in NSCs leads to increase the global DNA methylation and delayed neurodegeneration [27]. Recent evidences showed that Methyl CpG binding domain protein 1 (MBD1) is expressed in neural stem cells (aNSCs) of dentate gyrus of the adult hippocampus and maintains the integrity and stemness of NSC by inhibiting differentiation [28]. MBD1 and Methyl CpG binding protein 2 (Mecp2) belong to the methyl-CpG-binding protein family and play a key role to link DNA methylation and transcriptional regulation on differentiation genes [29]. MBD1 deficiency leads to the accumulation of undifferentiated NSCs and impaired transition into the neuronal lineage [28]. DNA methylation is closely related to stem cell-related diseases. A recent study found that there are a large number of gene mutations of DNMT3A in acute myeloid leukemia which is a malignant tumor characterized by clonal stem cell proliferation and aberrant block in differentiation [30]. Fetal alcohol syndrome showed that alcohol exposure to cultured NSCs altered normal DNA methylation programming of key neural stem cell genes and retarded NSC migration and differentiation [31], supporting the role of aberrant patterns of DNA methylation in fetal neural development after embryonic alcohol exposure.

### 2.2. Histone Modification

Histone modification refers to the process of histone methylation, acetylation, phosphorylation, polyadenylation, ubiquitination, and ADP glycosylation under the action of related enzymes. Histone-mediated epigenetic gene silencing is to remove acetyl groups from histone tails catalyzed by histone deacetylase (HDAC) enzymes and enhance the binding of histones to DNA and the aggregation of chromosomes, preventing transcription factors into the regulatory region [32]. HDAC1 is highly expressed in the oligodendrocyte differentiation period of the corpus callosum; HDAC inhibitors blocked oligodendrocyte differentiation and cause demyelination in the corpus callosum of postnatal rats [33]. The recent study indicated that the Arf-p53 axis also might be involved in the regulation of histone acetylation on the proliferation and senescence of the neurospheres [34].

Histone demethylation is also an important histone modification. It has two families, LSD1 (Lysine-specific demethylase 1) and JmjC (a domain), to regulate the proliferation and differentiation of stem cells. Inhibiting the activity of LSD1 or knockdown of LSD1 expression leads to the decreased proliferation of neural stem cells [35]. In addition, LSD1 plays a crucial role in maintaining the silencing of several developmental genes in human embryonic stem cells by regulating the balance between H3K4 (lysine 4 on histone H3 protein) and H3K27 methylation in its regulatory region [36]. Thus, histone modifications play a role in inducing NSC differentiation into neurons and glial lineages, but the mechanisms are still not clear.

### 2.3. Noncoding RNA

Noncoding RNAs (ncRNAs) are a class of RNA molecules that have no ability to translate into proteins but function as regulatory factors at transcriptional or posttranscriptional levels, including ribosomal RNAs (rRNAs), microRNAs (miRNAs), piwi-interacting RNAs (piRNAs), long noncoding RNAs (lncRNAs), and others [37]. These ncRNAs have shown to play distinct but also conserved roles in regulation of differentiation of NSCs [38–40]. Among different ncRNAs, current evidences demonstrate that miRNAs play critical roles in the regulation of differentiation of NSCs. miRNAs are a group of small RNA molecules of 20–24 nucleotides widely found in eukaryotes. They bind to target mRNAs to regulate their gene expression by promoting the degradation of target mRNAs. Similarly, microRNA is also involved in the regulation of NSC differentiation and proliferation dynamic homeostasis; for example, high levels of miR-184, which are inhibited by methyl-CpG binding protein 1, promote stem cell proliferation but inhibit adult neural stem/progenitor cell (aNSCs) differentiation [41]. MiR-145 directly regulates Nurr1 (a nuclear receptor) expression level, and overexpression of miR-145 inhibits the differentiation effect of BMP2; knockdown of miR-145 promoted the upregulation of Nurr1, resulting in the differentiation of NSCs into dopaminergic neurons [42]. MicroRNA can regulate many factors such as CT4, SOX2, and KLF4 in embryonic stem cells that are the direct targets of miR-145. The deletion of miR-145 increases the expression of OCT4, SOX2, and KLF4 and further inhibits the differentiation of NSC [43]. Recent studies showed that aging process begin when hypothalamic stem cells that coexpress Sox2 and Bmi1 are ablated accompanying with substantial loss of hypothalamic cells; the injection of exosomal miRNA in the cerebrospinal fluid, greatly prevented the cell aging process [44]. Therefore, more and more evidences showed that ncRNAs like miRNA are involved in the regulation of differentiation of NSCs.

## 3. Ten-Eleven Translocation (Tet) Proteins-5hmC Modification-Related Enzymes

Tet family proteins are a group of *α*-ketoglutarate (*α*-KG) and Fe2+ ‐dependent monooxygenase to catalyze the conversion of 5mC to 5hmC, concluding Tet1, Tet2, and Tet2 [45]. In 2009, Tahiliani et al. found that Tet1 catalyzes the reaction of 5mC to 5hmC [46]; thereafter, Tet2 and Tet3 have been found to have similar catalytic activity [47]. Although the main functions of the three enzymes are to oxidize 5mC to 5hmC, the distribution of the enzymes is different. The expression of Tet1 protein in embryonic stem cells and nervous system is high [48–50]; Tet2 is widely distributed and relatively high in hematopoietic system; Tet3 is mainly expressed in colon, muscle tissues, and less in brain tissues [51]. The three Tet enzymes contain a structurally similar carboxyl terminal catalytic region, which catalyzes the synthesis of 5hmC activity [45]. The catalytic domain of Tet proteins has 3 metal ion (Fe2+) and *α*-KG binding site to enhance its catalytic activity [46]. Tet1 and Tet3 have an amino terminal CXXC zinc finger protein domain, whereas Tet2 lacks this structure and needs to be assisted by IDAX protein with similar functions [52]. The CXXC domain protein of Tet2 is encoded by a distinct gene IDAX. The IDAX CXXC domain binds DNA sequences containing unmethylated CpG dinucleotides, localizes to promoters and CpG islands in genomic DNA, and interacts directly with the catalytic domain of Tet2 [52]. IDAX (also known as CXXC4), a reported inhibitor of Wnt signaling, regulates Tet2 protein expression [53]. Unexpectedly, IDAX expression results in caspase activation and Tet2 protein downregulation in a manner that depends on DNA binding through the IDAX CXXC domain, suggesting that IDAX recruits Tet2 to DNA before degradation [52]. Notably, the IDAX-related protein CXXC5 resembles IDAX in inhibiting Wnt signaling [54]. Therefore, the distribution and structure of Tet enzymes determine the distribution of 5hmC modifications in brain and their different roles in different diseases. Tet1 knockout mice showed impaired hippocampal neurogenesis resulting in learning and memory deficiency [55]. Tet2 functional disruption or knockout influences hematopoietic cell homeostasis and hematopoietic differentiation and promotes the development of myeloid malignancies [56]. Although either Tet1 or tet2 knockout mice are viable and fertile, Tet3 knockout mice are perinatally lethal [51]. These demonstrate the different roles of Tet proteins in the different tissues and in the devolvement of different organs. The functions of Tet proteins and its related phenotypes in rodent animals and diseases in human are summarized in Table 1.

5-Hydroxymethylcytosine (5hmC), the oxidative product of 5mC, was found in mammals with surprisingly high abundance in 2009 [46, 57]. Recent studies showed that 5mC is not the final chemical steps for gene silencing; Tet protein-associated DNA demethylation can transform 5-methyl cytosine (5mC) into 5-hydroxymethycytosine (5hmC), 5-formylcytosine (5fC), and 5-carbosycytosine (5caC), but 5fC and 5caC is much less than 5-hydroxymethylcytosine [58, 59]. Interestingly, for individual tissues, the levels of 5hmC, 5fC, and 5caC were not significantly related; for example, although 5hmC is more abundant in mouse brains than in ESCs, the levels of 5fC and 5caC are less abundant [45]. 5fC and 5caC can be further removed by the base excision repair (BER) pathway and thymine-DNA glycosylase [60]. This pattern suggests that the different steps of demethylation cycle are different in different tissues [61]. In addition, Tet protein overexpression or depletion can increase or decrease the content of 5hmC, 5fC, and 5caC in the genome [61]. The discovery of Tet proteins speeds the exploration of the functions of 5hmC [46, 57]. Because of its important functions, 5hmC in DNA has been considered as the sixth base. More evidences show that demethylation by 5hmC regulates the proliferation of NSCs and neurogenesis [27]. Therefore, we further discuss the regulation of 5hmC on NSCs and related neurological diseases.

## 4. Tet Proteins and DNA 5hmC Modifications Are Involved in the Regulation on the Proliferation and Differentiation of NSCs

The direction of cell differentiation is determined by the specific expression of tissue-specific genes, while DNA 5mC is involved in the regulation of gene expression and differentiation of cells in a specific direction [62–64]. Previous studies have shown that about 1.4% of CpG islands undergo a significant remethylation process during the differentiation of embryonic stem cells into NSCs and NPCs [65]. The increasing line of evidences indicate that 5mC directly inhibits transcription factors to bind to DNA [23] or recruits MeCP2 and MBD to form a complex and further prevent gene transcriptions that relate to the differentiation of NSCs [66]. Therefore, DNA methylation plays an important role in neural cell differentiation. Apparently, as an important demethylation mechanism, DNA 5hmC modification and Tet enzymes can be involved in the regulations of NSCs in theory.

Recently, 5hmC has been found in the mammalian genome and has been shown to be about 10 times more abundant in neurons than in some peripheral nervous tissues [67]. This suggests that 5hmC may be a stable epigenetic marker involved in cell specific mechanisms to achieve its function in the brain. More and more evidences demonstrated that Tet enzymes and Tet-mediated 5hmC modifications are involved in the proliferation and differentiation of ESCs and NSCs [29, 58, 68, 69]. Hahn et al. found that the increase of 5hmC modification in gene bodies is associated with genes important for neuronal functions during neuronal differentiation in mouse brain regions; however, gene activation for neuronal differentiation is not related to substantial DNA demethylation [69]. At the same time, overexpression of Tet2 and Tet3 also promotes the progression of neuronal differentiation [69]. Similarly, in Sirt6-knockout ESCs, the expression of Oct4, Sox2, and Nanog (the downstream of Sirt6) is inhibited and the upregulation of Tet enzymes and the significant increase of DNA 5hmC are found, resulting in ESC skewed development towards neuroectoderm [68]. This suggests that Sirt6-regulated ESC differentiation is in a Tet enzyme and 5hmC-dependent manner [68], supporting Hahn et al.'s results. A recent study further demonstrates that 5hmC dynamics is correlated with the differentiation of aNSCs; however, Tet2 primarily contributes to 5hmC acquisition during the differentiation of aNSCs [58]. Therefore, these evidences support the critical role of 5hmC modifications in the differentiation of NSCs.

**Figure 1:**
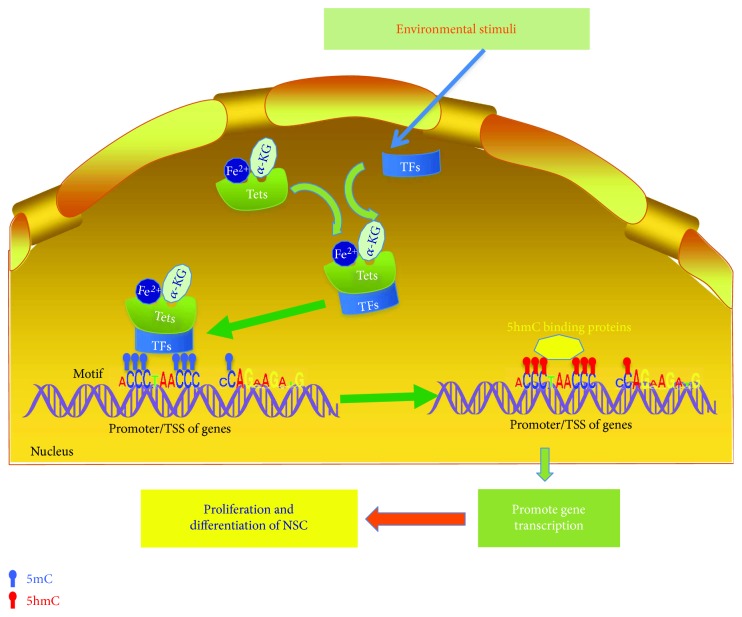
Tet proteins and 5-hmC mediated regulation of NSC proliferation and differentiation. Under the conditions of environmental stimuli, some transcriptional factors (TFs) such as FOXO3a enhance the affinity to Tet proteins along with cofactors of Tet enzymes including *α*-KG and Fe^2+^ to form a functional complex. By binding to DNA motifs of the targeting genes, the TFs guide the Tet enzymes to catalyze the conversion of 5mC to 5hmC. Generation of 5hmC facilitates the recruitment of the 5hmC binding proteins or other factors to enhance the transcription of targeting genes, thereby regulating the proliferation and differentiation of NSCs.

Tet proteins, as the important enzymes for the conversion of 5mC to 5hmC, also showed their functions on the proliferation/differentiation of NSCs. Tet1 depletion impairs hippocampal neurogenesis accompanied with poor learning and memory in mice; at the same time, Tet1 deficiency results in reduced neural progenitor pool in adult subgranular zone [55]. These results provided in vivo evidences that Tet1 deficiency in the central nervous system decrease the proliferation of adult NSCs in the hippocampal dentate gyrus. Moran-Crusio et al. showed that the depletion of Tet2 stimulates aNSCs proliferation but impairs the differentiation of aNSCs [56]. Tet2 interacted with the neuronal transcription activator Foxo3a, a member of the helix-turn-helix-like family proteins [70], and coregulated key genes involved in aNSC differentiation [58]. Moreover, Tet3 plays critical roles in neural progenitor cell maintenance [71] but is not required for NSC fate [72]. However, how Tet proteins interact with cofactors to regulate target genes responsible for the proliferation and differentiation of NSCs remains unclear. The possible regulative mechanisms are proposed in Figure 1.

## 5. Abnormal 5hmC and Neurological Diseases

The growing evidences demonstrate that 5hmC has high abundance in the brain and play a critical role to in the maintenance of normal neurodevelopment and functions of central nervous system. Thus, accumulating evidences showed that abnormal 5hmC modifications are involved the pathophysiology of different neurological diseases.

### 5.1. Alzheimer's Disease (AD)

Alzheimer's disease (AD) is one of the most common age-related neurodegenerative disorders in the central nervous system, characterized by progressive cognitive decline and loss of neuronal cells [73]. The pathogenesis of AD has yet to be defined, but there are evidences to support its genetic abnormalities, such as the mutations in *β*-amyloid precursor gene and presenilin1/2. Previous study has shown that AD is associated with DNA methylation [74]. It has been found that levels of 5mC and DNMT in neurons are reduced in patients with AD [74]. At the same time, 5hmC level was reported to decrease in the hippocampal tissue of patients with AD [75]. However, a study has shown that brain 5mC and 5hmC levels increased in patients with AD [76]. The reasons for this inconsistency need to be further investigated. In APP-presenilin1 double transgenic mice, 5hmC abundance in different brain regions showed differential response to the pathogenesis [77]. Further gene ontology analyses indicated that differential hydroxymethylation region- (DhMR-) associated genes are highly enriched in multiple signaling pathways involving neuronal development/differentiation [77], suggesting that DNA 5hmC modification is an epigenetic modifier on neurogenesis or NSC differentiation in aging or AD [78]. Interestingly, Tet1 is found to decrease in the hippocampus of patients with AD [79]. Tet1 knockout mice show impaired hippocampal neurogenesis as well as learning and memory defects [55, 80]. Therefore, Tet1 functions as a critical enzyme to regulate 5hmC modifications on those genes related to the proliferation and differentiation of NSCs and further promotes neurogenesis in adult brains.

### 5.2. Huntington's Disease (HD)

HD is an autosomal dominant disorder characterized by chorea, dystonia, slow and unexpected decline in cognitive function, and mental disorders [81]. At present, Huntington gene exon CAG repeats are considered as the major cause that leads to abnormal accumulation of the first amino acid polyglutamine in huntingtin proteins. Despite extensive research, the pathogenesis of neurodegeneration in HD is still unknown. ADORA2A gene encodes an adenylate A2A receptor, a G protein-coupled receptor that is highly expressed in the normal basal ganglia and is severely reduced in HD [82]. Recent studies have shown that HD results in an increase of 5mC expression and a decrease of 5hmC expression at the 5′-UTR end of the ADORA2A gene compared with the same age group [83]. Except for the decreased of ADORA2A gene 5hmC modification, a significant decrease of global 5hmC modification is found in HD mice with 128 CAG repeats, indicating the involvement of 5hmC in the pathogenesis of HD and a novel epigenetic marker in HD [82]. Further 5hmC profiling analysis indicates that most genes with differentially hydroxymethylated regions are highly related to the pathological changes in HD, suggesting that gene 5hmC modifications are involved in the regulation of neurogenesis, neuronal function, and survival in HD brain [82]. Because previous studies have shown the abnormal neurogenesis in HD [84], aberrant epigenetic regulation on relevant genes may impair the neurogenesis in brains with HD. Recent study demonstrated that targeting histone modification to downregulate the key genes for the pathology of HD causes beneficial effects in a Drosophila model of HD [85]. Therefore, the modulation of 5hmC signature in HD may be an effective strategy to ameliorate the symptoms of HD.

### 5.3. Rett Syndrome

Rett syndrome is considered as an inherited disease characterized by progressive mental decline, autistic behavior, ataxia, and anxiety in the early life of those who suffer from the disease. The etiology and genetic pattern of this disease remain unknown. The primary cause of Rett syndrome is caused by methyl CpG binding protein 2 (MeCP2) gene mutations that result in loss of function of MeCP2 [86]. Because brains have the highest expression of MeCP2, MeCP2 functional deficiency causes neurological diseases such as Rett syndrome [87]. Recent study showed that MeCP2 was identified as the major 5hmc binding protein in the brain to facilitate gene expression by organizing the chromatin [88]. Previous study showed a reverse correlation between MeCP2 and 5hmC level, suggesting that MeCP2 binds to 5mC blocking the conversion of 5mC to 5hmC [67]. MeCP2 mutations such as R133C (an MeCP2 residue mutated in Rett syndrome) preferentially abolish its binding ability to 5hmC and account for the role of 5hmC in the pathophysiology of Rett syndrome, supporting that 5hmC and MeCP2 constitute an epigenetic regulation complex to control cell differentiation or chromatin structure [88]. Recent studies have shown that MeCP2 is required for brain development and neuronal differentiation by inhibiting the ID1/Her2 (the zebrafish ortholog of mammalian Hes5) axis in zebrafish because genetic depletion of MeCP2 inhibited neuronal differentiation but its overexpression promoted neuronal differentiation [89]. However, it is still unclear whether the blocking of MeCP2 binding to 5hmC is responsible for neuronal differentiation in Rett syndrome, as awaits more investigations.

### 5.4. Major Depressive Disorders (MDD)

The high morbidity and suicide of depression has become a major health concern in the world [90]. However, the pathogenesis of MDD remains unclear. So far, genetic and environmental factors are considered to interact and participate in the MDD, in which environmental factors mainly affect gene transcription and expression through epigenetic modification. DNA methylation is considered a major epigenetic modification from environmental stress [91]. 5hmC functions as a new DNA demethylation mechanism, however, its role in depressive disorders is unclear. Epigenetic 5hmC modification, to some extent, provides a possible mechanism for explaining environmental factors that affect gene expression. Recent reports showed that patients with MDD had decreased gray matter volume and white matter integrity in the hippocampus [92]. In addition, Bansal et al. found structural changes in the cerebral cortex of patients with MDD, indicating that thickening of the cerebral cortex is a compensatory nerve growth response [93]. Recent studies have also provided evidence that Tet1 knockout showed antidepressive phenotypes by affecting neurogenesis in the hippocampus [94]. Therefore, Tet proteins-mediated 5hmC modifications on depression-related genes are involved the regulation of neurogenesis in the mechanisms of MDD.

## 6. Conclusions

Epigenetic modification is likely to be the collective response to changes in environmental factors as a means of cells or organisms to mitigate the adverse effects [95]. The dynamic changes of methylation (5mC) and demethylation (5hmC) in DNA could affect its structure as well as the functions of genes and further lead to different kinds of diseases. Recent advances on 5hmC modification have demonstrated that Tet proteins and Tet-mediated 5hmC play important roles in the proliferation and differentiation of NSCs. However, it is unclear how Tet protein, Tet-interacting factors, and DNA 5hmC in target genes interplay and regulate the devolvement of NSCs. These need more investigations in the future. Recently, DNA N6-Methyldeoxyadenosine (6 mA) is emerging as a new DNA modification and plays an important role in the regulation of the proliferation and differentiation of NSCs [96–98]. The interaction or crosstalking of DNA 5hmC modification and 6 mA modification will be an interesting topic. Considering the critical role of neuronal stem cells in the neurological diseases, targeting epigenetic regulation, especially on DNA 5hmC modification, is a promising strategy for the treatment of these neurological diseases.

## Figures and Tables

**Table 1:** Tet proteins and their functions.

Genes	Distribution	Structure	Functions of Tet enzymes	
			Knockout phenotypes in rodents	Related diseases in humans
Tet1	Mainly in ESCs and nervous system [48].	Contains CXXC, Cys-rich, and DSBH domains	(1) Abnormal hippocampal neurogenesis, with learning and memory fading [55]. (2) Antidepressive phenotypes [94] (3) Skews differentiation towards extraembryonic lineages in the teratoma [99].	(1) Acute leukemia [100]. (2) Gastric cancer ([101, 102], Deng, [103]). (3) Breast cancer [104].

Tet2	Widely distributed and high in hematopoietic system [48].	Contains Cys-rich and DSBH domains without CXXC domain	(1) Hematopoietic cell homeostasis and hematopoietic differentiation impairment, myeloid malignancies [56]. (2) Retinal neurons developmental failure in zebrafish [105].	(1) Polycythemia vera [106, 107]. (2) Primary myelofibrosis [107]. (3) Myelodysplastic syndrome [106]. (4) Myeloproliferative neoplasm [108]. (5) Melanoma [109].

Tet3	Mainly in colon and muscle tissues, less in brain tissue [51].	Contains CXXC, Cys-rich, and DSBH domains	(1) Developmental failure [72] and embryonic sublethality [110]. (2) Impaired differentiation and increased apoptosis [72]. (3) Fear extinction impairments in mice [111]. (4) Abnormal morphogenesis of retinal neurons in zebrafish [105]. (5) Abnormal neural differentiation and skewed toward cardiac mesodermal fate in mouse ESC [112].	NA
Tet1/2 DKO			Embryonic stage death and little normal growth [113]	NA
Tet1/3 DKO			(1) Dendritic arborization inhibition in mice [114] (2) Holoprosencephaly [115].	NA
Tet1/2/3 TKO			Developmental disorders [116]	NA

CXXC: Cys-X-X-Cys domain; DSBH: double-stranded beta helix; DKO: double knockout; ESCs: embryonic stem cells; TKO: triple knockout; NA: not available.
